# Supplementary material for: A Humanized Yeast Phenomic Model of Deoxycytidine Kinase to Predict Genetic Buffering of Nucleoside Analog Cytotoxicity
Source: Genes (Basel). 2019 Sep 30;10(10):770. doi: 10.3390/genes10100770 (PMC6826991; doi:10.3390/genes10100770)
Supplement: Supplementary file 1 [file genes-10-00770-s001.zip › Additional_Files/Additional_File7_GOTermHeatmaps.zip!/Additional_File7_GOTermHeatmaps/Arp2_3_protein_complex.pdf]

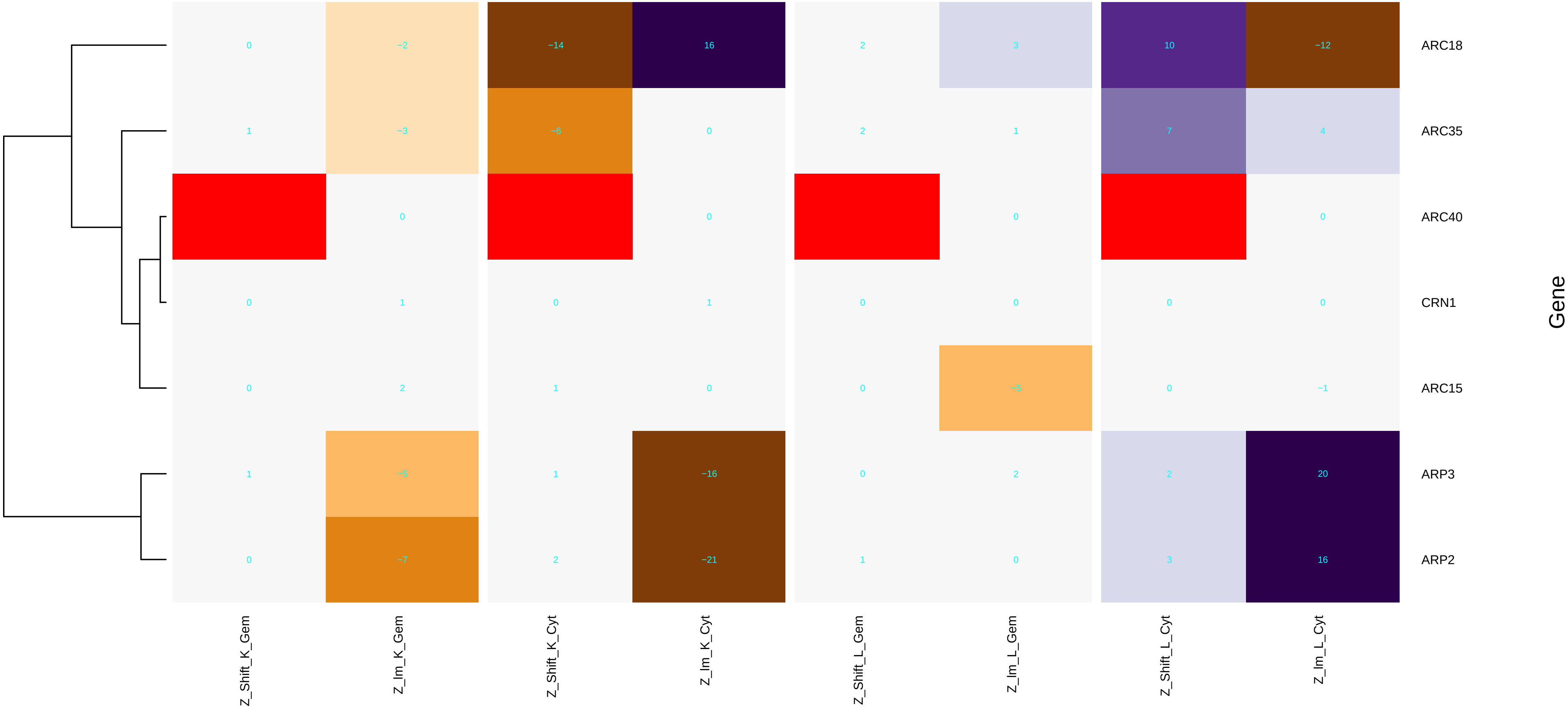

ARC18
ARC35
ARC40
CRN1
ARC15
ARP3
ARP2
Gene
Z_Shift_K_Gem
Z_Im_K_Gem
Z_Shift_K_Cyt
Z_Im_K_Cyt
Z_Shift_L_Gem
Z_Im_L_Gem
Z_Shift_L_Cyt
Z_Im_L_Cyt
0 −2 −14 16 2 3 10 −12
1 −3 −6 0 2 1 7 4
0 0 0 0
0 1 0 1 0 0 0 0
0 2 1 0 0 −5 0 −1
1 −5 1 −16 0 2 2 20
0 −7 2 −21 1 0 3 16
